# The Denser Canopy of Mangrove Drives the Structure of Insect Communities

**DOI:** 10.21315/tlsr2020.31.3.6

**Published:** 2020-10-15

**Authors:** Yendra Pratama Setyawan, Wakhid, Suhadi

**Affiliations:** 1 Pest and Entomology Section, Crop Protection Department, Smart Research Institute, Jl. Teuku Umar 19, Pekanbaru 28112, Riau, Indonesia; 2 Program of Entomology, Graduate School, IPB University, Jl. Meranti, Kampus IPB Darmaga, Bogor 16680, Indonesia; 3 Department of Biology, Faculty of Mathematics and Natural Sciences, State University of Malang, Jl. Semarang 5, Malang 65145, East Java, Indonesia

**Keywords:** Abundance, Canopy Cover, Diversity Index, Herbivores Insect, Mangrove Restoration

## Abstract

Mangrove restoration in Trenggalek, East Java has resulted an age variation of mangrove ecosystem. Diverse species of insects predominantly found in mangroves were collected using yellow pan traps, swipe nets and by direct picking from three different sites. This research was conducted from April until August 2015. There are 9,181 individual insects associated with mangroves comprised of 42 species from 31 families and eight orders. The first site or the 15 years old mangrove (66.22% canopy cover) indicated the highest Shannon diversity index at 2.54, Evenness index of 0.32 and Margalef richness index of 4.84. The lowest diversity was recorded in the third site or the five years old mangrove (19.65% canopy cover), with the Shannon diversity index at 2.28, Evenness index at 0.26 and Margalef richness index at 4.59. The most abundant species located was the *Eristena mangalis*, with 1,724 individuals (relative abundance of 18.78%), followed by *Monolepta* sp. with 1,649 individuals (relative abundance of 17.96%). These are the phytophagous insects associated with mangrove leaves. This study concluded that the older mangrove ecosystem have a denser canopy that supports insect life.

**Highlights**

- Three areas of mangrove restoration have different tree height and canopy cover, the older mangrove has the highest tree and denser canopy significantly compared to young mangrove.
- *Eristena mangalis* (Lepidoptera: Pyralidae), *Monolepta* sp. (Coleoptera: Chrysomelidae) and *Ryparida wallacei* (Coleoptera: Chrysomelidae) were dominant insects in mangrove ecosystem.
- The structure community of insects in the denser canopy was higher compared to young mangrove.

## INTRODUCTION

Since mangrove destruction started, efforts are being made to conserve mangrove as they are valuable for social life and extremely beneficial for ecosystems (Brander *et al*. 2012; Kerry 2017; Kusmana 2018). Mangroves can occupy the intertidal area, they interact with aquatic and terrestrial ecosystems, which helps to support diverse flora and fauna under mangrove vegetation (Macintosh & Ashton 2002; Romanach *et al*. 2018; Martin *et al*. 2019), one of which is insects. Insects play a crucial role in ecology and provide a strong linkage between the mangrove ecosystem and round ecosystems (Anneboina & Kavi 2017).

Insects can be permanent residents or temporary visitors to the mangrove ecosystem and might play as a pest (Faridah-Hanum *et al*. 2014; Srinivasan *et al*. 2014). They are herbivores that feed on leaves, flowers, seeds, stem or mangrove propagules (Macintosh & Ashton 2002; Ong *et al*. 2010). Honeybees are also associated with mangrove as they can be helpful, since the mangrove apiculture is an activity in economic productivity (Hill & Webster 1995; Ong *et al*. 2010; Fernandes *et al*. 2012; Kusmana 2018). Furthermore, some insects play crucial roles as pollinators that can maintain the ecosystem by ensuring reproduction (Macintosh & Ashton 2002; Chen *et al*. 2018).

Insects play an important role in nutrient flow and biochemical energy in the mangrove ecosystem. The high abundance of insects in mangroves confirms mangrove litter detritus formation and ecosystem function (Rahman 2002; Cannicci *et al*. 2008; Nagelkerken *et al*. 2008). This insect plays a significant role in detritus production processes and energy flow in the inshore mangrove system (Fleming *et al*. 1990; Rahman 2002). Furthermore, the turnover rate of nutrients and organic sedimentation are also increased by the attack of mangrove insects on the leaves and stems (Macintosh 1991; Rahman 2002; Krauss *et al*. 2008). The abundance of insects in the colonies of dead mangrove tree trunks and fallen timber increase the detritus formation (Ng & Sivasothi 2002; Rahman 2002; Nagelkerken *et al*. 2008).

Mangrove insects have been studied at some locations in Southeast Asia (Rahman 2002; Rahmawaty *et al*. 2005; Ong *et al*. 2010; Yatin *et al*. 2015). Further study in other locations need to be performed to provide necessary information related to the insect in the mangrove ecosystem. Mangrove ecosystems in Trenggalek, East Java, Indonesia are constantly under pressure due to various human activities, such as farming and agriculture. They have an impact on the exploitation of existing biological resources, which is the destruction of the mangrove ecosystem. The activities of preserving mangrove area have been carried out in the last few years by planting some species of mangrove.

The planting of mangroves in several locations has resulted in age variations of the mangrove ecosystem in Trenggalek. Therefore, it is necessary to conduct a study that focused on the insect structure in a different age of mangrove. The parameters used to describe the insect structure are abundance and diversity of insects. It will be useful for gaining basic information for future research focused on the diversity of insects in mangrove and further conservation management of the mangrove ecosystem. Furthermore, it will be the evidence of the relationship between insect diversity and canopy percentage. The results of abundance and diversity of insects should be different depending on the age of mangrove and canopy density.

## MATERIALS AND METHODS

### Study Site and Determination of Sampling Unit

This study was conducted from April until August 2015 at the mangrove area in Trenggalek region (−8.3068715, 111.7062334) which is under the management of Marine and Fishery Office of Trenggalek, East Java, Indonesia. There were three different mangrove ages, 15 years old (first site), eight years old (second site) and five years old (third site) (Syaifudin 2010). Tree height is also measured as a support data for mangrove age (Kalliovirta & Tokola 2005; Altman *et al*. 2016). Each site is separated by 5 km distance. A total number of 15 mangrove species exist in this location: *Rhizophora apiculata, Rhizophora mucronate, Terminalia catappa, Scyphyphora hydrophyllacea, Heritiera littoralis, Bruguiera cylindrical, Sonneratia alba, Lumnitzera racemosa, Xilocarpus muloccensis, Lumnitzera littorea, Avicennia lanata, Avicennia marina, Ceriops tagal, Ceriops decandra* and *Exocaria agalocha* (Syaifudin 2010).

A total of 15 plots (10 m × 10 m for each plot) were assigned in each site. The highest tree from each plot will be used as a centre tree. The average distance among plots was 50 m. These plots are placed by using purposive random sampling and arranged to cover each site. The tree height, canopy density and insect sampling will also be measured in the same site.

### Tree Height and Canopy Measurement

Thirty (30) trees selected from each site (two trees from each plot) will be used for tree height and canopy measurement. The tree height is measured using indirect techniques. An indirect technique is the most commonly used for standing trees (Larsen *et al*. 1987) because the tip is often inaccessible. Using a range finder calibrated in meters, the distance from a squatting position to the highest point on the tree crown was measured. The distance from the same spot to the tree base was also measured with a measuring tape (Leverett 2010; Hunter *et al*. 2013). Based on the Pythagoras Theorem of right-angled triangles, the tree height was finally computed.

C=(a2-b2)×12

where;

a = the distance to the highest point on the tree (hypotenuse)
b = the distance to the tree base
C = the tree height

The canopy is measured using densiometer for 30 trees (the same tree used for tree height measurement). Four observations are made for each tree and the accurate duplication of the observer procedure must be assured (Williams *et al*. 2003). The densiometer holding it at breast height. The canopy density is estimated by counting how many of 24 squares on a mirrored grid are covered by image of the canopy. The curved mirror reflects the canopy above and canopy closure. During each observation, the observer should try to: (i) hold the instrument level, (ii) keep it oriented in the desired direction, and (iii) hold a certain viewing angle so as to prevent parallax of the reflection angle between the eye and the mirror (Nuttle 1997; Korhonen *et al*. 2006).

### Sampling Techniques and Insect Identification

Insects are collected by using a yellow pan trap, swipe net and direct picking in all sites twice in a month. The yellow pan trap (Ø 20 cm) are installed into each plot by 50 cm from ground (15 pan traps per site). Each pan trap filled by water and detergent to break the water surface. All pan traps are modified with a netting on the site to prevent overflow during heavy rainfall. The traps installation started at 07:00 am and collected three days later. Insects explorations are also conducted by using swipe net every 3 h (started 07:00 until 16:00) in the same plot (15 plots 10 m × 10 m per site explored by swipe net and direct picking). Sometimes the plant material needed to be sampled by cutting the stems and branches with plant cutters because some insects such as wood-borers insect can only be found in the stems or branches (Kandasamy 2003; Ong *et al*. 2010). The insect captured in this study will be placed in ethanol 70% and brought to Ecology laboratory at State University of Malang for further identification. Samples were identified from the lowest taxonomic level following some insect identification key based on morphological character (Murphy 1989; Commonwealth Scientific and Industrial Research Organisation (CSIRO) 1991; Ping *et al*. 2003; Braby 2011; Yen 2012; Schnitzler *et al*. 2014).

### Data Analysis

All the data were calculated using Microsoft Excel. The difference of tree height and canopy cover was tested using analysis of variance (ANOVA). Insect diversity for each site was analysed using Shannon index (H′), Evenness index (E′) and Richness index (R) and calculated using Margalef Index in R statistical software. The estimated curve to visualise the collected species number is calculated using PAST version 3 software.

## RESULTS

### Tree Height and Canopy Cover of Mangrove

Mangrove ecosystem in Trenggalek has different tree height and canopy cover based on mangrove age (Table 1). In this study, the older mangrove has the highest tree (F_2, 87_ = 646.74, *P* < 0.001) and denser canopy compared to the youngest mangrove (F_2, 87_ = 402.76, *P* < 0.001). Mangrove in the first site (15 years old) has the highest tree (8.36 m) and denser canopy (66.22%) followed by the second site or 8 years old mangrove with 4.68 m tree height and 30.51% for canopy cover and 5 years old mangrove has the lowest tree height (2.26 m) and canopy cover (19.65%).

### The Insect Diversity and Abundance

**Figure 1:**
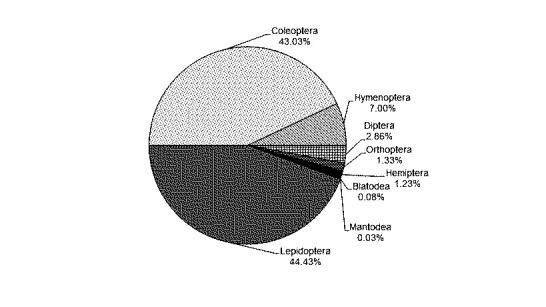
The percentage of each order from captured insect.

This study found 9,181 individual of insects associated with mangrove, consisting of 42 species from 31 families and 8 orders (Table 2). Lepidoptera was the dominant species (44.43%), followed by Coleoptera (43.03%), Hymenoptera (7%), Diptera (2.86%), Orthoptera (1.33%), Hemiptera (1.23%), Blatodea (0.08%) and Mantodea (0.03%) (Fig. 1). Species with the most abundant insects was *Eristena mangalis* (Lepidoptera: Pyralidae) with 1,724 individuals (18.78%), followed by *Monolepta* sp. (Coleoptera: Chrysomelidae) with 1,649 insects (17.96%) and *Rhyparida wallacei* (Coleoptera: Chrysomelidae) with 1,262 insects (13.75%).

**Figure 2:**
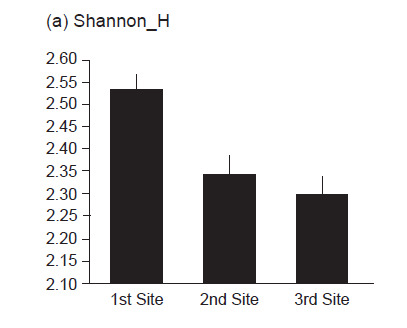

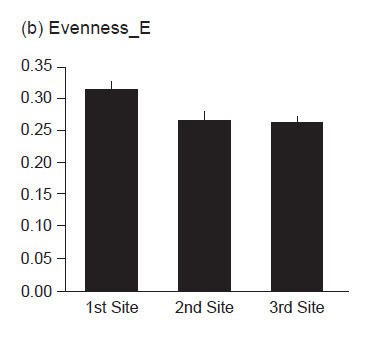

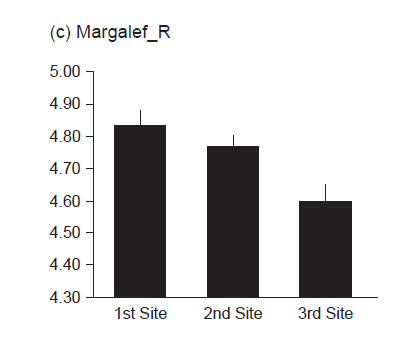
Comparison of insect diversity index measured by (a) Shannon diversity index, (b) Evenness index, and (c) Margalef richness index.

**Figure 3:**
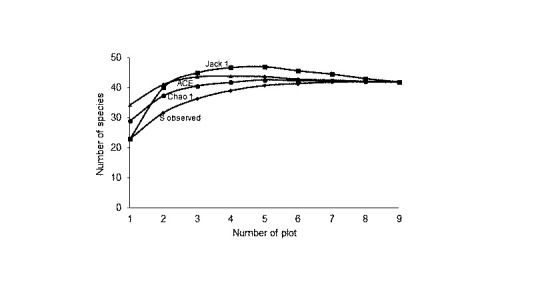
Estimates of insects on mangrove ecosystem based on number of plots.

The number of species and individual that were found in the first site (40 species and 3,169 individuals) were higher compared to the second (39 species and 2,889 individuals) and the third site (37 species and 9,181 individuals). The three sites showed high insect diversity (H′ > 2.0); first site (H′ = 2.54), second site (H′ = 2.34), and third site (H′ = 2.29). Additionally, the first site accounts for the highest value for Shannon diversity index (H′ = 2.54), Evenness index (E′ = 0.32), and Margalef richness index (R′ = 4.84) (Fig. 2). The estimation of insect species demonstrate an increase in the number of species in plot 1 to plot 4. This shows that a large number of insect species were found during the sampling period. Then, in the fifth plot, the estimated curve reached the asymptote point, and there was no curve increase until the end of the plot (Fig. 3). Achieving the estimator curve at the asymptote point shows that the observed mangrove insects are well collected. In addition, the estimation curve that reaches the asymptote point is suspected because there are no rare species (Rubio *et al*. 2008).

## DISCUSSIONS

The mangroves ecosystem provides habitat and resource that supports a large number of insects at different trophic levels (Faridah-Hanum *et al*. 2014). Insects perform many vital functions. They aerate the soil, pollinate blossoms and control insect and plant pests. The primary trophic groups are herbivorous insects that feed on leaves, wood borer, and flower/fruit/seed-feeding (Cannicci *et al*. 2008; Doydee *et al*. 2010); saproxylic and saprophagous insects feed on dead and decaying organic material (Fleming *et al*. 1990), and parasitic and predatory insects feed or prey on other animals (Nagelkerken *et al*. 2008).

Most of the insects in this study are Lepidopteran and Coleopteran. Most of them are herbivore insects that feed on leaves and stem borer. Other studies also mention that both orders become dominant insect at mangrove ecosystem in Alas Purwo National Park, East Java (Astri *et al*. 2018) and Gili Meno, North Lombok (Yatin *et al*. 2015). The larvae of common aquatic moth, *Eristena mangalis* – a moth of the Pyralidae family, feed on young leaves (Macintosh 1991; Nagelkerken *et al*. 2008). The larvae of *Odites* sp., known as mangrove moth, are found on the foliage of various species of Rhizophoraceae (Kandasamy 2003). One of the most common leaf-eating beetles in the mangrove is *Rhyparida wallacei* (Macintosh 1991). Another beetle that is dominant in mangrove is *Monolepta* sp. beetle, which can cause extensive leave damage, especially on Avicennia mangrove (Kandasamy 2003; Faridah-Hanum *et al*. 2014). Among the insects, ants play an important ecological role. Their high abundance and multitude of interactions are engaged in making them essential for ecosystem functioning (Wilson 1959; Cannicci *et al*. 2008). Ants are able to protect plants against herbivores via their predatory and territorial behaviour (Bronstein 1998; Styrsky & Eubanks 2007).

The species found in this study were more diverse compared to different locations in Indonesia. Astri *et al*. (2018) reported that 12 species from 9 families were associated with mangrove in Alas Purwo National Park, East Java. Eighty arboreal insects were found in Kupang bay, East Nusa Tenggara (Tokan *et al*. 2018). Ashuri *et al*. (2016) also reported that 32 insects were found in the ecosystem of mangrove in Wonorejo, Surabaya. Another result from outside Indonesia, the study in Singapore reported that 16 species found and *Monolepta* sp. beetle becomes a serious pest in mangrove in Singapore (Lim *et al*. 2001). The bark beetles, *Dendroctonus* sp. found in this study is *D. frontalis* while the bark beetle found in Tamil Nadu, India is *D. mican* (Srinivasan *et al*. 2014). Furthermore, the leaf webbing caterpillar, *Odites* sp. were also reported attacking all the species of *Aviciennia* spp. in Tamil Nadu, India (Kandasamy 2003).

This research has found that the first site has a greater diversity index compared to others. This may be because the first site, which is the oldest of the three ecosystems, has a denser canopy typical (Cannicci *et al*. 2008), resulting in a greater contrast in light levels (Tokan *et al*. 2018). Vertical structure complexity increases with successional age (Humphrey *et al*. 1999). In addition, Intachat *et al*. (1999) also reported that mangroves could be expected to have lower herbivore diversity than other types as a result of their lower dense canopy.

Some studies have mentioned that the tree canopy supports the highest diversity of insect. The study from Simmon and Linsenmair (2001) in Malaysia mentions that the density of herbivorous insects associated with the canopy of *Qurcus subsericea* is greater in the dense canopy compared to the lower canopy, suggesting that these microenvironments were appropriate with the insect communities. On the other hand, the number of herbivorous insect was higher in the understory than in canopy (Murakami *et al*. 2007; Levey *et al*. 2016), meaning that the environmental factors such as light can explain this result (Rego *et al*. 2019).

The results from this study indicate that diverse species of insects are found in a mangrove in Trenggalek, East Java. Mangroves provide a habitat that supports a large number of insects at different trophic levels (Nadkarni 2001; Valencia-Cuevas & Tovar-Sánchez 2015). Further study will help us to identify the new species of coastal environmental insects and analyse the same factors that can support insect life. On the other hand, insects and plants are becoming extinct because of habitat loss, over-exploitation, pollution, overpopulation and global climate change (Macintosh & Ashton 2002; Kannan & Padmanaban 2013; Yatin *et al*. 2015). Hence it will be necessary to conduct a further detailed study, including seasonal surveys and other methods, to counteract these challenges by investigating the insects and other factors in this area for biodiversity conservation and management.

## CONCLUSION

Mangrove ecosystem is approved as a suitable ecosystem for insect. The diverse species of insects found in this study area, where 9,181 individual insects of 42 species from 31 families and 8 orders associated with mangroves. The mangrove ecosystem on the first site (15 years old) has higher canopy cover percentage (66.22%) that resulting height insect diversity compared to second site (8 years old) and third site (5 years old). It becomes the evidence that mangrove ecosystems have a relatively old age and dense canopy that can support more variety of insects.

## Figures and Tables

**Table 1:** Tree height and canopy cover comparison for each site.

Location^a^	Number of samples	Tree height (m)^b^	Min–max height (m)	Canopy cover (%)	Min–max canopy cover (%)
First site	30	8.36^a^	7.16–9.87	66.22^a^	46.58–78.21
Second site	30	4.68^b^	3.28–6.01	30.51^b^	22.35–39.14
Third site	30	2.26^c^	1.09–3.41	19.65^c^	7.23–32.40

*Notes*:

a Three site with different mangrove age. First site is 15 years old mangrove, second site is eight years old mangrove and third site is three years old mangrove

b The tree height and canopy cover percentage that followed by different letter indicated significantly different based on DMRT (95%)

**Table 2:** The number of insects associated with mangrove based on a different age.

Order	Family	Insects species	Number of species per site			∑	%

			First site	Second site	Third site		
Blatodea	Blattidae	*Blatta lateralis*	1	5	1	7	0.08
Coleoptera	Curculionidae	*Dendroctonus frontalis*	120	194	140	454	4.94
	Attelabidae	*Rhynchites* sp.	81	71	52	204	2.22
	Cerambycidae	*Aeolesthes holosericeus*	47	76	37	160	1.74
	Chrysomelidae	*Monolepta* sp.	506	173	970	1649	17.96
	Chrysomelidae	*Rhyparida wallacei*	289	628	345	1262	13.75
	Scolytidae	*Coccotrypes rhizophorae*	85	85	52	222	2.42
Diptera	Calliphoridae	*Lucilia sericata*	2	1	1	4	0.04
	Culicidae	*Aedes egipty*	11	23	20	54	0.59
	Muscidae	*Musca domestica*	49	39	37	125	1.36
	Syrphidae	*Eristalinus* sp.	3	1	2	6	0.07
	Syrphidae	*Allograpta* sp.	23	18	29	70	0.76
	Tephritidae	*Elleipsa* sp.	2	1	1	4	0.04
Hemiptera	Alydidae	*Leptocorisa* sp.	2	2	0	4	0.04
	Cicadellidae	*Cicadella viridis*	2	2	3	7	0.08
	Pseudococcidae	*Pseudococcus comstocki*	37	35	15	87	0.95
	Scutelleridae	*Calliphara excellens*	2	2	1	5	0.05
	Pentatomidae	*Murgantia histrionica*	1	1	0	2	0.02
	Pentatomidae	*Calliphara* sp.	2	1	5	8	0.09
Hymenoptera	Apidae	*Apis indica*	10	9	9	28	0.30
	Apidae	*Xylocopa violacea*	1	2	0	3	0.03
	Calcididae	*Calcididae* sp.1	3	1	1	5	0.05
	Calcididae	*Calcididae* sp.2	2	1	0	3	0.03
	Formicidea	*Atta sexdens*	319	77	40	436	4.75
	Formicidea	*Atta* sp.	62	32	55	149	1.62
	Formicidea	*Plectroctena* sp.1	3	2	1	6	0.07
	Formicidae	*Plectroctena* sp.2	1	0	1	2	0.02
	Ichneumonidae	*Ichneumonidae* sp.	2	0	1	3	0.03
	Apidae	*Xylocopa virginica*	0	3	1	4	0.04
	Vespidae	*Polistes fuscatus*	2	1	1	4	0.04
Lepidoptera	Noctuidae	*Autoba alabastrata*	231	121	183	535	5.83
	Pyralidae	*Eristena mangalis*	357	899	468	1724	18.78
	Phyllocnistidae	*Phyllocnistis* sp.	86	101	56	243	2.65
	Tortricidae	*Eupoicillia* sp.	134	89	187	410	4.47
	Xyloryctidae	*Odites* sp.	645	152	370	1167	12.71
Mantodea	Mantidae	*Archimantis* sp.	0	1	2	3	0.03
Orthoptera	Acrididae	*Patanga japonica*	7	8	14	29	0.32
	Acrididae	*Omocestus viridulus*	10	15	10	35	0.38
	Tettigoniidae	*Eulophophyllum* sp.	3	1	3	7	0.08
	Pirgomorphydae	*Atractomorpha crenulata*	8	8	3	19	0.21
	Pirgomorphydae	*Atractomorpha lata*	17	8	5	30	0.33
	Gryllidae	*Apteronemobius* sp.	1	0	1	2	0.02

Insect abundant per site		3,169	2,889	3,123	9,181	100.00	
